# An insecticide resistance-breaking mosquitocide targeting inward rectifier potassium channels in vectors of Zika virus and malaria

**DOI:** 10.1038/srep36954

**Published:** 2016-11-16

**Authors:** Daniel R. Swale, Darren W. Engers, Sean R. Bollinger, Aaron Gross, Edna Alfaro Inocente, Emily Days, Fariba Kanga, Reed M. Johnson, Liu Yang, Jeffrey R. Bloomquist, Corey R. Hopkins, Peter M. Piermarini, Jerod S. Denton

**Affiliations:** 1 Department of Anesthesiology, Vanderbilt University Medical Center, Nashville, TN 37232, USA; 2 Department of Entomology, Louisiana State University Agricultural Center, Baton Rouge, LA, 70803, USA; 3 Department of Pharmacology, Vanderbilt University, Nashville, TN 37232, USA; 4 Vanderbilt Center for Neuroscience Drug Discovery, Vanderbilt University Medical Center, Nashville, TN 37232, USA; 5 Department of Entomology and Nematology, University of Florida, Gainesville, FL 32610, USA; 6 Department of Entomology, Ohio Agricultural Research and Development Center, The Ohio State University, Wooster, OH 44691, USA; 7 Vanderbilt Institute of Chemical Biology, Vanderbilt University Medical Center, Nashville, TN 37232, USA; 8 Department of Chemistry, Vanderbilt University, Nashville, TN 37235, USA; 9 Institute for Global Health, Vanderbilt University, Nashville, TN 37203, USA.

## Abstract

Insecticide resistance is a growing threat to mosquito control programs around the world, thus creating the need to discover novel target sites and target-specific compounds for insecticide development. Emerging evidence suggests that mosquito inward rectifier potassium (Kir) channels represent viable molecular targets for developing insecticides with new mechanisms of action. Here we describe the discovery and characterization of VU041, a submicromolar-affinity inhibitor of *Anopheles (An.) gambiae* and *Aedes (Ae.*) *aegypti* Kir1 channels that incapacitates adult female mosquitoes from representative insecticide-susceptible and -resistant strains of *An. gambiae* (G3 and Akron, respectively) and *Ae. aegypti* (Liverpool and Puerto Rico, respectively) following topical application. VU041 is selective for mosquito Kir channels over several mammalian orthologs, with the exception of Kir2.1, and is not lethal to honey bees. Medicinal chemistry was used to develop an analog, termed VU730, which retains activity toward mosquito Kir1 but is not active against Kir2.1 or other mammalian Kir channels. Thus, VU041 and VU730 are promising chemical scaffolds for developing new classes of insecticides to combat insecticide-resistant mosquitoes and the transmission of mosquito-borne diseases, such as Zika virus, without harmful effects on humans and beneficial insects.

Mosquitoes are vectors of numerous human pathogens that impose enormous health and socioeconomic burdens on the developing world. The malaria vector *An. gambiae* and the dengue/yellow fever vector *Ae. aegypti* are collectively responsible for hundreds of millions of cases of malaria and dengue fever annually, leading to over 500,000 deaths per year^1^,^2^,^3^. Moreover, *Ae. aegypti* is suspected as the primary vector in the recent outbreak of Zika virus in Latin America and the Caribbean; Zika virus has been causally linked to dramatic increases in the number of cases of microcephaly and Guillan-Barré syndrome^4^,^5^. The two major classes of insecticides used in vector control programs are pyrethroids and anticholinergics (i.e., carbamates/organophosphates). These agents work, respectively, by blocking inactivation of voltage-gated sodium channels or inhibiting acetylcholinesterase enzymes expressed in the nervous system^6^,^7^,^8^. Moreover, they act on all developmental stages and sexes, creating intense selective pressure for target site resistance (e.g., knockdown resistance, *kdr*) and/or metabolic resistance (e.g., elevated expression of cytochrome P450 monoxygenases)^9^,^10^,^11^. The development of resistance and lack of novel, validated target sites that can be exploited for mosquito control complicate efforts to mitigate the spread of emerging mosquito-borne pathogens that have recently circumscribed the globe, such as Zika and chikungunya viruses. Thus, new classes of insecticides acting on novel molecular targets are needed to bolster integrated vector control programs and limit the spread of mosquito-borne diseases.

*R*ecent genetic and pharmacological evidence suggests that inward rectifier potassium (Kir) channels could represent viable targets for new mosquitocides. In *Drosophila melanogaster*, embryonic depletion of Kir1, Kir2, or Kir3 mRNA levels leads to death or defects in wing development^12^. Knocking down Kir1 and Kir2 mRNA expression in the heart and Malpighian (renal) tubules of *Drosophila*, respectively, inhibits the immune response against cardiotropic viruses^13^ and transepithelial secretion of fluid and K^+^ 14. In yellow fever mosquitoes (i.e. *Ae. aegypti*), pharmacological inhibition of Kir1 with structurally-distinct small molecules (i.e. VU573, VU590, or VU625) disrupts the secretion of fluid and K^+^ in isolated Malpighian tubules, and impairs flight, urine production, and K^+^ homeostasis in intact females^15^,^16^,^17^,^18^. A major limitation of the potential use of these inhibitors as adulticides is their inability to penetrate the mosquito cuticle, thereby requiring microinjection to induce toxicity. Thus, one of our goals is to identify Kir1 inhibitors that can kill and/or incapacitate mosquitoes after topical application. Another goal is to identify inhibitors that are specific for mosquito Kir channels over mammalian Kir channels, since the latter play fundamental roles in *nerve, muscle, endocrine, and epithelial cell function*^19^.

In the present study, we employed high-throughput screening to identify a Kir1 inhibitor, termed VU041, which exhibits similar toxicity to adult female mosquitoes from representative insecticide-susceptible and -resistant strains of *An. gambiae* (G3 and Akron, respectively) and *Ae. aegypti* (Liverpool and Puerto Rico, respectively). Moreover, topical VU041 application to adult female mosquitoes of both species inhibits their fecundity. Importantly, VU041 is selective for mosquito Kir channels over mammalian Kir channel orthologs and non-lethal to adult honey bees (*Apis mellifera*). Thus, VU041 represents a promising chemical scaffold for the development of a new generation of insecticides to control mosquitoes without harmful effects on humans and beneficial insects.

## Results

### Discovery of VU041

**Figure 1:**
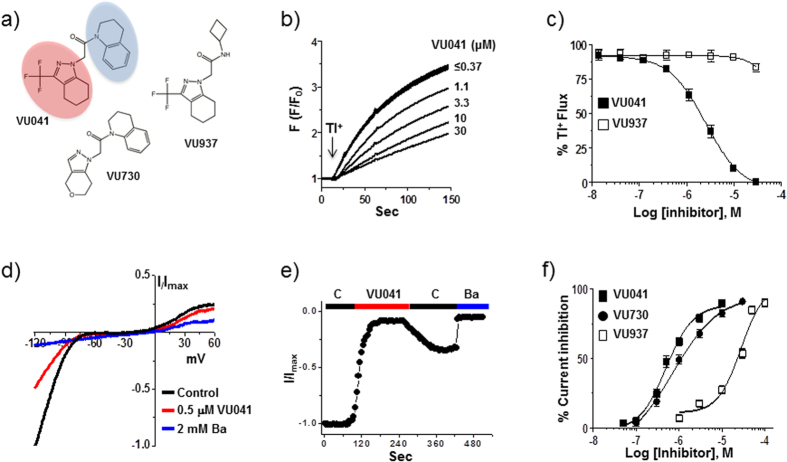
Design and characterization of *An*Kir1 small-molecule inhibitors. (**a**) Modular approach to assess two areas of diversification of VU041 through a medicinal chemistry campaign: heterocyclic portion (red shading) and the dihydroquinoline portion (blue shading) of the molecule. See text for details. (**b**) Representative fluorescence traces showing dose-dependent inhibition of the *An*Kir1-mediated Tl^+^ flux by VU041 with concentrations ranging from 0.12 to 30 μM. The arrows indicate the addition of extracellular Tl^+^. (**c**) Concentration-response curves (CRCs) for VU041 and VU937 derived from Tl^+^ flux assays. The IC_50_ and Hill-coefficient values for VU041 are 2.5 μM (95% CI: 1.9–3.3 μM) and 1.1, respectively. The IC_50_ for VU937 was not determined due to less than 50% inhibition at 30 μM. Data are mean ± SEM; n = 5 independent experiments performed in triplicate. (**d**) Representative current-voltage plots of *An*Kir1 current in the presence of control saline, 0.5 μM VU041, and 2 mM Ba (positive control). (**e**) Time-course and reversibility of VU041-dependent inhibition of *An*Kir1 heterologously expressed in T-Rex-HEK293 cells. The cells were voltage clamped at a holding potential of −75 mV, stepped to −120 mV and then ramped to +120 mV every five seconds before returning to −75 mV. After current stabilization in control (C) saline, the superfusate was switched to one containing 10 μM VU041 until steady inhibition was reached. After this, VU041 was washed out of the bath in exchange for control (C) saline to allow wash-out. Barium (Ba) was added at the end of each experiment to block remaining *An*Kir1 current. (**f**) CRCs of VU041, VU730, and VU937 derived from patch clamp experiments (n = 4–6) against heterologously expressed *An*Kir1 cells.

Approximately 26,000 compounds were screened for pharmacological modulators of *An*Kir1 channel activity, leading to the discovery of 121 confirmed *An*Kir1 inhibitors. We focused on 1-(3,4-dihydroquinolin-1(2*H*)-yl)-2-(3-(trifluoromethyl)-4,5,6,7-tetrahydro-1*H*-indazol-1-yl)ethan-1-one (termed ‘VU041′ hereafter, Fig. 1a), because of its 1) potent inhibition of *An*Kir1-dependent thallium (Tl^+^) flux *in vitro* (e.g., Fig. 1b,c, Table S1) and 2) high partition coefficient (cLogP > 4), making it likely to penetrate the mosquito cuticle^20^.

In whole-cell patch clamp experiments (Fig. 1d–f), VU041 inhibited *An*Kir1 with an IC_50_ of 496 nM (95% CI: 396–619 nM; Hill coefficient value of 1.3), making it the 2^nd^ most potent *in vitro* inhibitor of mosquito Kir1 channels discovered to date; VU625 remains the most potent *in vitro* inhibitor (IC_50_ ~ 100 nM), but is not topically toxic to mosquitoes which prevents practical use^16^. *An*Kir1 inhibition by VU041 was rapid and partially reversible (Fig. 1e). Two-electrode voltage clamp experiments in *Xenopus* oocytes revealed that VU041 preferentially inhibited *Ae*Kir1 over *Ae*Kir2B, both of which are expressed in the Malpighian tubules (Fig. S2).

The selectivity of VU041 for mosquito vs. mammalian Kir channels was evaluated in quantitative Tl^+^ flux experiments against *An*Kir1, *Ae*Kir1, and a panel of Kir channels that play critical physiological roles in mammals: Kir1.1 (kidney), Kir2.1 (heart, brain), Kir4.1 (kidney, brain), Kir6.2/SUR1 (pancreas, brain), and Kir7.1 (broadly expressed). VU041 inhibited *An*Kir1 and *Ae*Kir1 with IC_50_ values of 2.5 μM and 1.7 μM, respectively. Importantly, VU041 inhibited mammalian Kir1.1, Kir4.1, Kir6.2/SUR1, and Kir7.1 by less than 10% at a concentration of 30 μM. The only mammalian Kir channel tested that VU041 inhibited appreciably was Kir2.1 (IC_50_ of 12.7 μM; Table S1).

### Lead optimization around the VU041 scaffold to enhance potency and mosquito selectivity

Lead optimization was initiated with the goal of improving the selectivity of VU041 for *An*Kir1 vs. Kir2.1. The first analog library was designed to keep the right-hand dihydroquinoline constant and evaluate the left-hand heterocyclic portion (Fig. 1a; red highlight). One compound (VU730, **5**) retained its activity toward *An*Kir1 (IC_50_ = 2.4 μM in Tl^+^ flux assays [Table S2]; IC_50_ = 717 nM in patch clamp experiments [Fig. 1f]), but lost activity toward Kir2.1 (IC_50_ > 30 μM in Tl^+^ flux assays [Table S2]). Our next library kept the left-hand trifluoromethyl tetrahydropyrazole constant while altering the right-hand amide portion of the molecule (Fig. 1a; blue highlight). Although none of the compounds in this series showed an increase in potency against *An*Kir1 (Table S3), VU937 (Compound **18**) inhibited *An*Kir1 channel activity in patch clamp experiments by 60-fold less than VU041 (IC_50_ = 29.7 μM; 95% CI: 17.7–49.9 μM) (Fig. 1f; Table S3). Due to the significant loss of potency, VU937 was used in subsequent experiments as an ‘inactive’ analog to confirm that any toxic or physiological effects of VU041 on mosquitoes were associated with its inhibition of Kir1.

### VU041 is equally toxic to insecticide-susceptible and -resistant strains of mosquitoes

**Figure 2:**
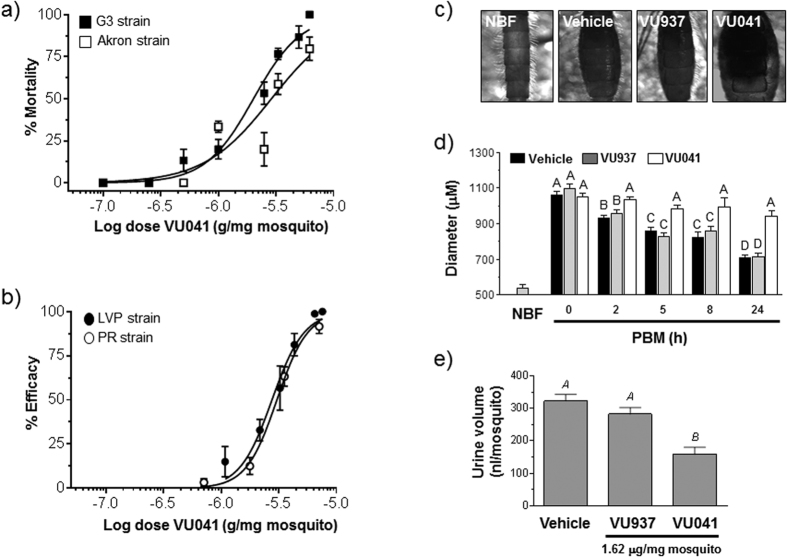
Toxicological characterization of VU041 in mosquitoes. (**a**) Toxicity of the susceptible (G3) and multi-resistant (Akron) strains of *An. gambiae* mosquitoes (adult females) 24 h after topical exposure to VU041 using n = 3 replicates of 30 mosquitoes per dose tested. (**b**) Toxicity of the susceptible (LVP) and pyrethroid-resistant (PR) strains of *Ae. aegypti* mosquitoes (adult females) 24 h after topical exposure to VU041 using n = 4–8 replicates of 10 mosquitoes per dose tested. (**c**) Still images showing mosquito (adult female *An. gambiae*, G3) abdomens 24 h post-blood meal; mosquitoes were treated topically with vehicle (negative control), VU937, or VU041 immediately after feeding. An abdomen from a non-blood fed (NBF) mosquito is shown for comparison. (**d**) diameters of mosquito abdomens during the first 24 h post-blood meal (PBM) after topical exposure to EtOH (n = 20), VU041 (n = 22), or VU937 (n = 18). Values are means ± SEM. Uppercase letters represent statistical significance as determined by a one-way ANOVA with a Tukey’s posttest (P < 0.05). (**e**) Amount of urine excreted by adult female *Ae. aegypti* (LVP) mosquitoes 1 h after injection with 900 nL of K^+^-PBS. Two hours prior to the injection, mosquitoes were treated topically with the vehicle, VU937 (1.7 μg/mg mosquito), or VU041 (1.7 μg/mg mosquito). Values are means ± SEM; n = 14, 8, and 11 trials of 5 mosquitoes each for the vehicle, VU937, and VU041 treatments, respectively. Upper-case letters indicate statistical categorization of the means as determined by a one-way ANOVA with a Newman-Keuls post-test (P < 0.05).

To determine if VU041 was topically toxic to mosquitoes, we applied the compound to the cuticles of insecticide-susceptible and insecticide-resistant strains of *An. gambiae* and *Ae. aegypti* (adult females) and assessed efficacy 24 h later. The resistant ‘Akron’ strain of *An. gambiae* is resistant to permethrin (33-fold) and propoxur (101-fold) when compared to the susceptible G3 strain of *An. gambiae* and is known to confer resistance through target-site (*kdr* and Modified AcetylCholine Esterase (MACE) and metabolic resistance mechanisms^21^,^22^,^23^,^24^. The resistant ‘Puerto Rico’ (PR) strain of *Ae. aegypti* possesses target-site (*kdr*) resistance (J.J. Becnel and BEI resources, personal communications), which contrasts from another Puerto Rican strain that possesses elevated mRNA levels encoding CYP450 enzymes^25^. Importantly, the ED_50_, or effective dose to incapacitate 50% of the mosquitoes, for VU041 was similar between the susceptible and resistant strains for each species (Fig. 2a,b; Table 1). In both species, VU937 was not toxic (Table 1, Fig. S3b), suggesting that the toxicity of VU041 was associated with its inhibition of Kir1 channels.

Consistent with VU041 eliciting similar efficacy in both strains of *An. gambiae*, pre-treatment of the susceptible (G3) strain with piperonyl butoxide (PBO), an inhibitor of cytochrome P450 monoxygenases (CYP450s), only enhanced the efficacy of VU041 by ~3-fold, whereas pre-treatment with *S,S,S*-tributyl phosphorotrithioate (DEF), an inhibitor of carboxyesterases, did not enhance toxicity (Table 1). Inhibition of CYP450s in the AKRON strain, which possess up to a 12-fold overexpression of some CYP450 genes^21^, enhanced toxicity 3-fold more than the G3 strain likely due to the increased levels of metabolic enzymes causing altered pharmacokinetics and pharmacodynamics in the resistant strain. Thus, VU041 is only moderately metabolized by cytochrome P450 enzymes and does not appear to be metabolized by esterases. Experiments in the G3 strain of *An. gambiae* with VU730, which does not inhibit mammalian Kir2.1, revealed a similar ED_50_ as that for VU041 (Table 1). Thus, VU041 is the first small-molecule inhibitor of mosquito Kir1 channels that exhibits topical toxicity in both insecticide-susceptible and -resistant lines of mosquitoes. Moreover, VU041 can be modified to reduce its inhibition of mammalian Kir2.1 without affecting its efficacy as a mosquitocide (e.g., VU730).

### VU041 inhibits renal excretory function in mosquitoes

A signature feature of inhibiting Kir channels in mosquitoes is impairment of fluid secretion/urine production in Malpighian tubules, which reduces the mosquito’s capacity for diuresis^15^,^16^,^17^,^18^. Diuresis plays an especially important role in adult female mosquitoes after a blood meal by excreting the excess fluid and electrolytes that are absorbed into the hemolymph^26^,^27^. We therefore evaluated whether VU041 disrupts fluid-volume regulation associated with blood meal processing in *An. gambiae* mosquitoes. Immediately after engorgement, mosquitoes were treated with an ED_30_ dose of VU041, and their abdominal diameters were measured over the following 24 h. In vehicle (control)- and VU937-treated mosquitoes, abdominal diameter increased approximately 2-fold immediately following blood feeding (Fig. 2c,d), and then decreased significantly over 24 h (Fig. 2c,d). In striking contrast, although the abdominal diameter of VU041-treated mosquitoes increased similarly, it did not change over the following 24 h period, consistent with VU041-dependent inhibition of fluid-volume excretion.

To directly determine whether VU041 impairs mosquito excretion, we performed an *in vivo* diuresis assay on adult female *Ae. aegypti*, using an approach we recently established for this species^15^,^16^, but the inhibitors were applied topically instead of injecting them into the hemolymph. The diuretic capacities of control and VU937-treated mosquitoes were similar to each other, whereas that of VU041-treated mosquitoes was significantly lower by ~51% compared to controls (Fig. 2e). Taken together with the data in Fig. 2c,d, these results suggest that VU041 impairs renal excretory function and fluid-volume regulation during blood meal processing in mosquitoes.

### VU041 reduces mosquito fecundity

**Figure 3:**
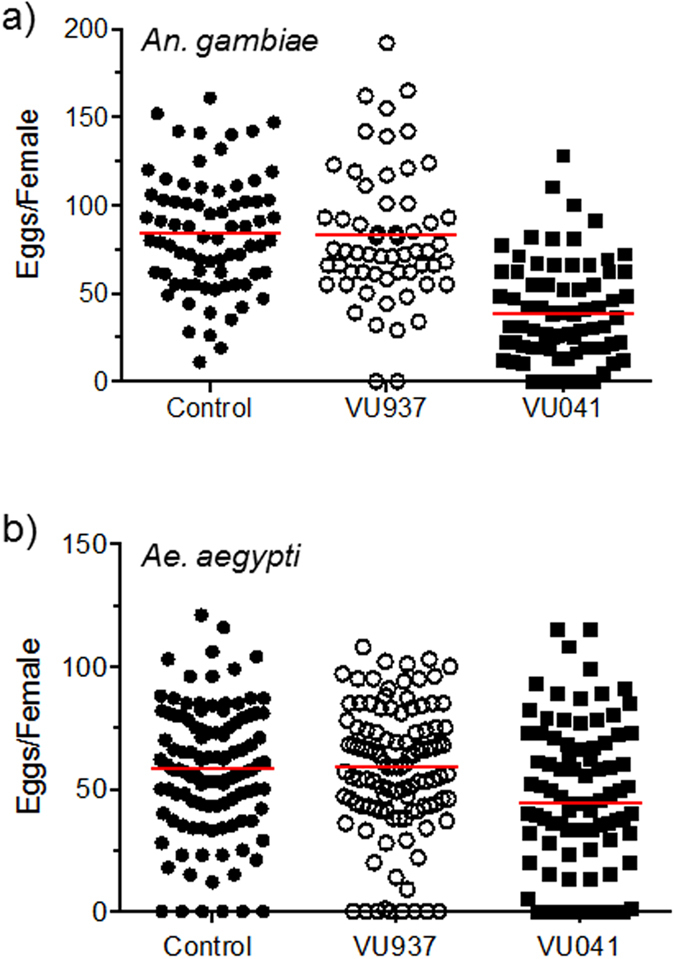
Effects of VU041 on mosquito fecundity. Number of eggs laid per female 72 h PBM for (**a**) *An. gambiae* and (**b**) *Ae. aegypti*. Mosquitoes were topically treated with the vehicle (control), VU937, or VU041 within 1 h following engorgement with blood. Each data point represents the egg output of an individual mosquito. Red bars indicate the median number of eggs laid for each treatment. The median number of eggs laid by VU041 mosquitoes was significantly lower than those laid for control and VU937-treated mosquitoes (P < 0.05), as determined by a Kruskal-Wallis ANOVA with a Dunn’s post-hoc analysis.

Given that VU041 disrupts blood meal processing and diuresis in mosquitoes (Fig. 2c–e), and that knock-down of *An*Kir1 expression via RNA interference reduces fecundity^28^, we hypothesized that VU041 would also reduce egg laying after blood feeding. Adult female mosquitoes of both species were topically treated with ~1 μg/mg mosquito (*An. gambiae*) or 3.4 μg/mg mosquito (*Ae. aegypti*) of VU041 or up to ~10 μg/mg mosquito (solubility limits) of VU937 within 1 h after engorgement, and the total number of eggs laid per mosquito were counted 72 h post blood feeding. For both *An. gambiae* and *Ae. aegypti*, the control and VU937-treated mosquitoes laid a similar median number of eggs per mosquito, whereas the VU041-treated mosquitoes laid a significantly lower median number of eggs per mosquito (Fig. 3). Thus, VU041 reduces mosquito fecundity.

### VU041 is not lethal to adult honeybees

**Figure 4:**
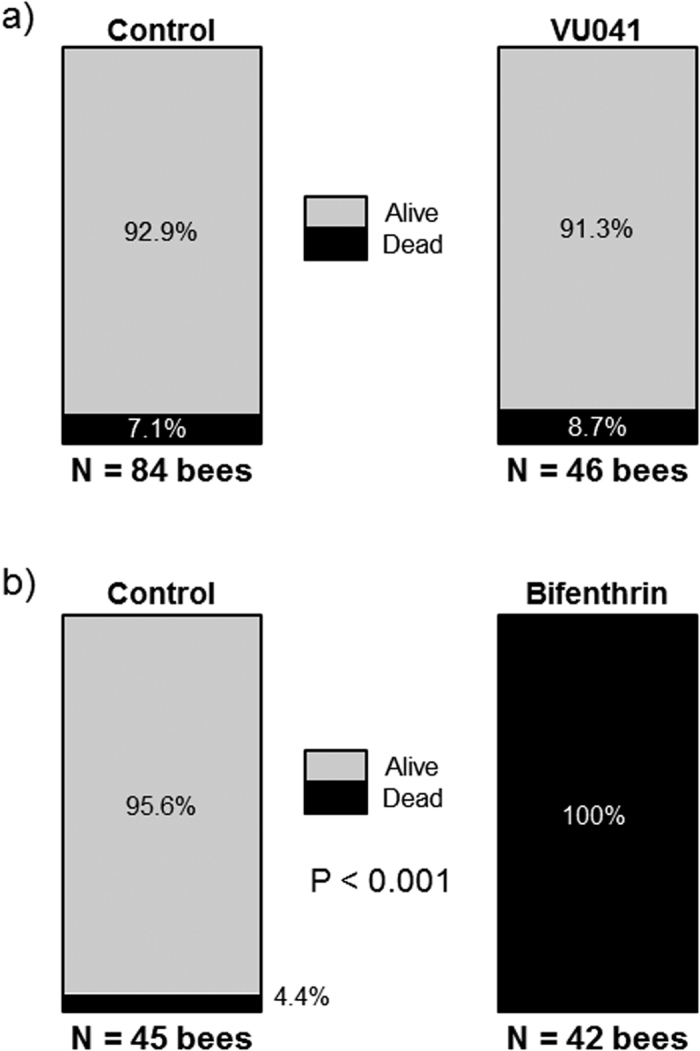
Toxicity of VU041 to the honey bee, *A. mellifera*. Percentage of bees alive (gray) or dead (black) 48 h after topical application of (**a**) solvent (control) or VU041 (1 mg per bee), or (**b**) solvent (control) or bifenthrin (0.3 μg per bee). P value indicates significant difference between control and treatment as determined by a Fisher’s Exact Test (P < 0.05).

Insecticide selectivity against pollinators is important given recent concerns over the role insecticides play in declines in pollinator health^29^. To determine if VU041 is toxic to honey bees, we treated 3-day old adult honey bees topically on the thoracic notum with a limit dose of VU041 (1 mg/bee; i.e., ~10 μg/mg), and assessed toxicity 48 h later compared to negative (vehicle) and positive (0.1 μg/bee bifenthrin) controls. As shown in Fig. 4, VU041 did not cause significant mortality to honey bees within 48 h compared to the vehicle(Fisher’s Exact Test, P = 0.74, N = 130), whereas application of bifenthrin resulted in 100% mortality at 48 h (P < 0.001, N = 87). Thus, VU041 is non-lethal to adult honey bees when applied topically.

## Discussion

Resistance of mosquitoes to conventional insecticides that target the nervous system, such as pyrethroids, carbamates, and organophosphates, is a major hurdle to the integrated management of mosquito-borne diseases^11^. As discussed below, the results of the present study provide compelling data that a small molecule inhibitor of mosquito Kir channels (VU041) offers a new active compound for the development of mosquitocides that overcome insecticide resistance and may also be safe to humans and pollinators.

### VU041 overcomes insecticide-resistance mechanisms

The Akron strain of *An. gambiae* used in the present study carries multiple resistance mechanisms including 1) mutations in a voltage-gated Na^+^-channel (*kdr*) that offers resistance to pyrethroids, 2) mutations in an AChE (MACE, ace-1R) that confers resistance to carbamates^30^,^31^,^32^,^33^, and 3) metabolic resistance derived from increased biochemical levels of CYP450s and carboxylesterases^21^. The Puerto Rican (PR) strain of *Ae. aegypti* used in this study is resistant to pyrethroids only through a point mutation (*kdr*) in the voltage-gated sodium channel.

*A priori*, one would not expect a mosquito strain with only target-site resistance in Na^+^ channels, such as the PR strain of *Ae. aegypti* used in this study, to exhibit resistance to a small molecule inhibitor of Kir channels. As such, VU041 showed similar efficacy against the LVP and PR strains of *Ae. aegypti*. However, mosquito strains with both target-site and metabolic resistance, such as the Akron strain of *An. gambiae*, may have a greater capacity to detoxify a small molecule, regardless of its molecular target. Importantly, VU041 showed similar efficacy against the Akron and G3 strains of *An. gambiae*, suggesting that VU041 is able to avert the elevated xenobiotic detoxification mechanisms of the Akron mosquitoes. Consistent with this finding, inhibition of CYP450s with PBO or esterases with DEF nominally enhanced the toxicity of VU041 against the G3 strain of *An. gambiae* (Table 1), suggesting that it is not efficiently metabolized by these important detoxifying enzymes. Inhibition of CYP450s in the Akron strain enhanced toxicity of VU041 at a greater level when compared to the G3 strain, presumably due to the significant overexpression of multiple CYP450 genes in this resistant strain. These data are consistent with VU041 being a modest substrate of this class of detoxification enzymes.

### Potential mechanisms/modes of action of VU041 on mosquitoes

Our *in vivo* experiments of blood meal processing and diuretic capacity suggest that one mechanism of action of VU041 is the disruption of excretory functions mediated by Malpighian tubules. This finding is consistent with previous studies by our group that indicate Kir channels are an important mechanism of transepithelial K^+^ and fluid secretion in mosquito Malpighian tubules, where they localize to the basolateral membrane of the epithelial cells^18^,^34^,^35^,^36^. The inhibition of Kir channels in Malpighian tubules is expected to disrupt the processing of blood meals by limiting the excretion of blood-derived electrolytes and water that are absorbed into the hemolymph. In addition, potential effects on the midgut’s digestion of blood and absorption of ions/fluid from the blood cannot be ruled out given that the mosquito midgut is a site of Kir mRNA expression and barium-sensitive K^+^ transport^28^,^37^,^38^. Our experiments in *An. gambiae* demonstrate that mosquitoes treated with VU041 retain a large abdominal girth 24 h after blood feeding compared to control mosquitoes, suggesting an impairment of blood meal processing, perhaps by disrupting the post-prandial diuresis and/or blood digestion. Our experiments in *Ae. aegypti* confirm that VU041 at least inhibits mosquito diuretic capacity, consistent with impairment of Malpighian tubule function as a contributor to the toxicity of this compound. This novel mechanism of action of VU041 on a physiological target outside of the nervous system may also contribute to its efficacy against the Akron and PR strains, which are resistant to neurotoxic carbamates and/or pyrethroids.

The combined effects of VU041 on blood-meal processing and diuresis may also contribute to its inhibitory effects on mosquito fecundity in *An. gambiae* and *Ae. aegypti*. That is, if VU041 inhibits blood meal digestion, nutrient absorption, and/or solute/metabolite excretion, then vitellogenesis may not proceed efficiently. Furthermore, a direct effect of VU041 on the ovaries cannot be ruled out. In a previous study on adult female *An. gambiae*, we demonstrated that Kir1 mRNA levels are enriched in the ovaries and Kir1 mRNA knockdown reduces fecundity^28^. Thus, the reduced fecundity observed in the current and previous study may be a combined effect of inhibiting Kir1 in the excretory and reproductive systems.

### Selectivity of VU041 for mosquito vs. mammalian Kir channels

The selectivity of new mosquitocides is of critical importance, because mosquito control methods often are implemented within or near human dwellings, especially in tropical regions with endemic malaria or dengue/Zika fever (e.g., aerial sprays, insecticide-treated bed nets). The results of our *in vitro* screening assays for VU041 show that this compound has a relatively clean ancillary pharmacology against a panel of mammalian Kir channels with no activity against Kir1.1, Kir4.1, Kir7.1, and Kir6.2/SUR1. However, VU041 moderately inhibits Kir2.1, which is highly expressed in the human heart; inhibition of this channel may have deleterious consequences on heart function^39^,^40^. Thus, we developed analogs of VU041 to determine if any structural changes led to increased selectivity for *An*Kir1 vs. Kir2.1. Remarkably, compound VU730 retained its inhibitor activity against *An*Kir1 without measureable inhibition of Kir2.1. Importantly, we confirmed that VU730 also retained its topical mosquitocidal toxicity with a similar potency as VU041. Although additional experiments will be necessary to demonstrate whether VU730 is non-toxic to mammals, our data suggest the exciting possibility that VU041 offers a chemical scaffold for the development of potent inhibitors of mosquito Kir channels with topical mosquitocidal activity and minimal inhibition of mammalian Kir channels.

### Selectivity of VU041 for mosquitoes vs. adult honey bees

The selectivity of new mosquitocides is also important to limit effects on beneficial insects, such as honey bees and other pollinators, which contribute to over $24 billion USD to the US economy (https://www.whitehouse.gov/the-press-office/2014/06/20/fact-sheet-economic-challenge-posed-declining-pollinator-populations). Recent studies have cited significant concerns over the effects of broad-spectrum insecticides (e.g., neonicotinoids, pyrethroids) on pollinator health^29^. Remarkably, a dose of 10 μg VU041 per mg adult honey bee (*A. mellifera*) was not toxic within 48 h. A similar dose in mosquitoes would exhibit ~100% efficacy within 24 h. The honey bee ortholog of Kir1 shares only ~55% amino acid identity with mosquito Kir1 channels^41^. Thus, the interaction of VU041 with mosquito Kir1 channels may occur in a domain that is not conserved with the bee Kir1 channel. Alternatively, the chemical composition of the honey bee cuticle may substantially differ from that of mosquitoes in a manner that reduces the penetration of VU041 into the hemolymph and/or the VU041 molecule may be more efficiently detoxified by bees relative to mosquitoes. Additional studies will be required to better understand the lack of activity in adult bees and to confirm that the compound is not toxic to adult and larval bees when added to their diets. Future studies will also be required to determine if VU041 has any chronic sub-lethal effects on the performance of honey bee colonies. However, the data of the present study at least provide the exciting possibility that VU041 could serve as scaffold for developing inhibitors of mosquito Kir channels that are toxic to mosquitoes with nominal effects on beneficial insects.

### Potential of VU041 for practical use against mosquitoes

Although VU041 represents a significant advance in the development of new mosquitocides targeting Kir channels (i.e., topical efficacy, potentially safe to humans and bees), it is important to note that this molecule requires additional chemical optimization and formulation development to improve its efficacy for use as a traditional mosquitocide in the field. However, the present study shows that VU041 impairs blood meal processing, renal function, and fecundity at relatively low, sub-lethal doses. Thus, if sub-lethal doses of Kir channel inhibitors can be delivered to adult female mosquitoes in the field, then we may expect to impair the longevitiy/fecundity of adult females instead of causing immediate death. As has been previously suggested, such an approach may dampen the propagation of resitance genes in a population and lead to the sustainable control of mosquito-borne diseases more effectively than traditional neurotoxic insecticides^42^,^43^.

To conclude, our study reports the first topically active, mosquito-selective, and resistance-mitigating small molecule inhibitor of a Kir channel. These data provide additional proof-of-concept and validation that Kir channels are viable insecticide target sites, as we have proposed in previous studies^15^,^16^,^17^,^18^. Future work will be aimed at increasing the *in vitro* and *in vivo* potency of these Kir channel inhibitors as well as fully elucidating their mechanisms of toxicity.

## Methods

### Stable T-REx-HEK293 cell line expressing Kir channels

The open-reading frame of a full-length cDNA encoding *An*Kir1 cloned from *An. gambiae* Malpighian tubules (Genbank Accession # KJ596497)^17^ was sub-cloned into the pcDNA5/TO expression vector (Life Technologies) and used for stable cell line generation, as described previously^44^,^45^. Expression constructs for mammalian Kir channels were developed and validated in previous studies by our group^46^,^47^,^48^.

### Chemical Synthesis and Lead Optimization

Methods for chemical synthesis are provided in the Supplemental Methods section.

### High Throughput Screening

Tl^+^ flux assays were performed essentially as described previously^17^,^44^,^47^. Briefly, stably transfected T-Rex-HEK-293 cells expressing *An*Kir1 channels were cultured overnight in 384-well plates (20,000 cells/20 μL/well black-walled, clear-bottomed BD PureCoat amine-coated plates (BD, Bedford, MA) with a plating media containing DMEM, 10% dialyzed FBS and 1 μg/mL tetracycline. Approximately twenty-four hours after cell plating, the cell culture medium was replaced with a dye-loading solution containing assay buffer (Hanks Balanced Salt Solution with 20 mM HEPES, pH 7.3), 0.01% (*w/v*) Pluronic F-127 (Life Technologies, Carlsbad, CA), and 1.2 μM of the thallium-sensitive dye Thallos-AM (TEFlabs, Austin, TX). Following 1 hr incubation at room temperature, the dye-loading solution was washed from the plates and replaced with 20 μL/well of assay buffer.

### Whole-cell patch clamp electrophysiology

Transiently transfected HEK-293T cells expressing *An*Kir1 cells were voltage clamped in the whole-cell configuration of the patch clamp technique, as described previously^17^,^47^,^48^. The extracellular bath solution contained (in mM): 135 NaCl, 5 KCl, 2 CaCl_2_, 1 MgCl_2_, 5 glucose, 10 HEPES free acid, pH 7.4, 290 mOsm/kg H_2_O. The pipette solution contained (in mM): 135 KCl, 2 MgCl_2_, 1 EGTA, 10 HEPES free acid, 2 Na_2_ATP (Roche, Indianapolis, IN), pH 7.3, 275 mOsm. Cells were voltage clamped at −75 mV, stepped to −120 mV for 200 msec, and then ramped to 120 mV at a rate of 2.4 mV/msec. Concentration-response curves (CRCs) were constructed by measuring the effects of increasing doses of inhibitors on *An*Kir1 currents at −120 mV. All recordings were made at room temperature (20–23 °C).

### Two-electrode voltage clamp electrophysiology

Heterologous expression of *Ae*Kir1 or *Ae*Kir2B was performed in *Xenopus laevis* oocytes as described previously^16^,^17^. Current-voltage (I-V) relationships of oocytes were acquired by clamping the membrane potential (V_m_) near the spontaneous, resting V_m_ and then initiating a voltage-stepping protocol (via the Clampex module of pCLAMP) consisting of 20 mV steps from −140 mV to +40 mV (100 ms each). Additional details are provided in the Supplemental Methods.

### Mosquito colonies

*An. gambiae* mosquitoes, G3 strain (MRA-112), were reared in an environmental chamber at 27 °C and 75% relative humidity at Vanderbilt University, Department of Biological Sciences, Nashville, TN. *An. gambiae*, Akron strain (MRA-913, isolated in Benin), was reared in a separate environmental chamber at the Emerging Pathogens Institute, University of Florida, Gainesville, FL at 27 °C and 75% relative humidity. The Akron strain of *An. gambiae* was selected every 5^th^ generation for anticholinergic and pyrethroid resistance by exposing adult mosquitoes to bendiocarb (12.5 μg/bottle) and permethrin (21.5 μg/bottle) using the CDC bottle assay. The survivors of each sex were then mixed and allowed to breed (personal communication, Mr. Paul Howell, Centers for Disease Control and Prevention, Atlanta, GA). Furthermore, to ensure resistance, the mosquitoes from each resistant egg cohort were exposed to an LC99 dose (based on G3 toxicity values) of permethrin and propoxur. The mosquitoes used in this study were derived from the same colony that was used to demonstrate the resistance of the Akron strain to propoxur and permethrin that is attributed to both target site and metabolic resistance through upregulation of CYP450 enzymes^21^,^22^,^23^,^49^.

An established colony of *Ae. aegypti* mosquitoes, Liverpool strain (LVP-IB12, MRA-735), was reared and maintained as previously described^15^ in an environmental chamber at 28 °C and 80% relative humidity at the Ohio Agricultural Research and Development Center (OARDC) of The Ohio State University, Wooster, OH. When needed, eggs from a pyrethroid-resistant strain of *Ae. aegypti*, Puerto Rico strain (PR, NR-48830), were obtained from BEI Resources, NIAID, NIH and reared to adults. The third-instar larvae of the resistant strain of *Ae. aegypti* were exposed to permethrin (0.1 mg/ml) every third generation to maintain the resistance trait (personal communication, Mr. Paul Howell, Centers for Disease Control and Prevention, Atlanta, GA). Adult mosquitoes of all strains were fed a 10% sucrose solution *ad libitum* and held under a 12 h/12 h light cycle. All experiments were carried out on adult females at 3–5 days post-eclosion.

### Toxicology experiments in An. gambiae

Topical toxicity bioassays were performed based on the method of Pridgeon *et al.*^50^. Briefly, non-blood fed adult female mosquitoes were chilled on ice for 1–3 minutes, during which 200 nL of chemical (dissolved in 95% ethanol) was applied onto the abdomen of the insect using a handheld Hamilton^®^ micro-applicator. For synergism studies, 500 ng of the synergist (i.e., piperonyl butoxide [PBO] or *S,S,S*-tributyl phosphorotrithioate [DEF]) per milligram of mosquito (ng/mg) was applied to the abdomen 4 h prior to application of the insecticide. For each compound, 6–8 doses that resulted in toxicity ranging between 0% and 100% were applied to a minimum of 30 mosquitoes each, and repeated 3 times on mosquito broods. The three ED_50_ values obtained were then averaged and presented as a mean. An ethanol-only treatment was included in each experiment as a negative control. Treated mosquitoes were transferred to small cages with access to 10% sucrose and held under rearing conditions for 24 h. Mortality was recorded at 24 h. Mortality data were pooled and analyzed by log-probit using Poloplus^®^ to determine 24 h ED_50_ values, after correcting for control mortality using Abbot’s formula^51^.

Blood meal processing studies were performed with similar methods as described above, with the exception that *An. gambiae* were blood fed on anesthetized mice. All methods were carried out in accordance with Vanderbilt Institutional Animal Care and Use Committee approval (protocol M/13/164 to Dr. Julian Hillyer, Department of Biology, Vanderbilt University). Upon completion of blood feeding, female mosquitoes with a fully distended abdomen were selected and 200 nL of chemical at a non-lethal concentration (1 μg/mg) was applied directly to the abdomen. Any mosquito that died during the 24 h observation period was excluded from the analysis. Images of the abdomens were acquired at 0, 2, 5, 8, and 24 h through the dorsal cuticle and were measured at the widest point of the abdomen. These images were captured using bright-field illumination on a Nikon 90i light microscope (Nikon Corp., Tokyo, Japan) connected to a Photometrics CoolSNAP HQ2 high-sensitivity monochrome CCD camera (Roper Scientific, Ottobrunn, Germany). Digital images were acquired using Nikon Advanced Research NIS-Elements software. Mean abdominal diameters were compared using a one-way ANOVA with a Tukey’s post-hoc analysis (Prism 6, Graphpad Software, La Jolla, CA).

### Toxicity experiments in Ae. aegypti

Topical toxicity bioassays in adult female *Ae. aegypti* (LVP and PR strains) were performed as described previously^52^. Briefly, for a given dose, 10 non-blood fed mosquitoes were immobilized on ice and 500 nL of VU041 was applied to the thorax of each using a handheld Hamilton^®^ microapplicator. A solvent-only treatment was included in each experiment as a negative control. Treated mosquitoes were transferred to small cages with access to 10% sucrose and held under rearing conditions for 24 h. The efficacy of a dose was measured as the percentage of treated mosquitoes in a cage that were flightless or dead at 24 h. Four to eight replicates of 10 mosquitoes were performed per dose. The ED_50_ values were determined using a non-linear curve fit analysis (log [inhibitor] vs. response variable-slope) in Prism 6 (Graphpad Software).

The efficacy of VU041 was compared to that of its inactive analog (i.e., VU937) only in LVP mosquitoes. In these experiments, two groups of 10 mosquitoes were treated with a dose of VU041 or VU937 near the ED_50_ of VU041 (3.24 μg/mg mosquito), and their condition was assessed 24 h later, as described above. Six replicate experiments of 10 mosquitoes were performed. The mean efficacies of the solvent, VU041, and VU937 were analyzed using a one-way ANOVA with a Newman-Keuls post hoc analysis (Prism 6, Graphpad Software).

### Diuresis experiments in Ae. aegypti

The excretory capacity of adult female *Ae. aegypti* (LVP strain) was measured as described previously^15^,^16^. In brief, groups of 5 mosquitoes were treated with a sub-lethal dose of VU041 (1.7 μg/mg mosquito), VU937 (1.7 μg/mg mosquito), or solvent 2 h before injecting the hemolymph of each mosquito with 900 nL of a potassium-enriched, phosphate-buffered saline (K^+^-PBS) using a Nanoject II microinjector (Drummond Scientific Company, Broomall, PA). The K^+^-PBS consisted of the following (in mM): 92.2 NaCl, 47.5 KCl, 10 Na_2_HPO_4_ and 2 KH_2_PO_4_ (pH 7.5). Each treatment group of 5 mosquitoes was transferred into a separate graduated, packed-cell volume tube (MidSci, St. Louis, MO) and held for 1 h at 28 °C. The volume excreted by the mosquitoes was measured visually via the graduated column at the bottom of the tube. At least 8 replicates (5 mosquitoes per replicate) were performed for each treatment. All mosquitoes were confirmed to be alive at the end of 1 h. The mean volumes excreted by solvent-, VU041-, and VU937-treated mosquitoes were analyzed using a one-way ANOVA with a Newman-Keuls post hoc analysis (Prism 6, Graphpad Software).

### Mosquito fecundity experiments

To determine the effects of VU041 on fecundity in *An. gambiae*, we used an assay similar to Raphemot *et al.*^28^ Briefly, adult female mosquitoes were given access to an anesthetized mouse for 60 min. After this time period, engorged mosquitoes were immobilized on ice and 200 nL of VU041 (ED_30_: 1 μg/mg of mosquito), VU937 (10 μg/mg of mosquito), or solvent was applied directly to their abdomens. After treatment with the respective drugs, individual female mosquitoes were transferred to *Drosophila* vials (Fisher Scientific, Pittsburg, OA) containing 2 mL of water. The total number of eggs were counted 72-hours after being transferred to the vial. Any mosquitoes that died during this 72-hour period were excluded from the analysis. All assays were performed in an environmental chamber that was maintained at 27 °C and 75% relative humidity and mosquitoes were given access to 10% sucrose solution *ad libitum*. At least 25 female mosquitoes were used per replicate for each treatment group; each treatment was repeated on three separate broods, giving a total number of individuals studied ranging from 75–113 for each group.

To determine the effects of VU041 on fecundity in *Ae. aegypti*, adult female mosquitoes were allowed to feed for 1 h on heparinized rabbit blood (Hemostat) presented in a membrane feeder (Hemotek). After the feeding period, the mosquitoes were immobilized on ice, visually inspected for blood engorgement, and topically treated with 500 nL of solvent, VU041 (3.4 μg/mg mosquito), or VU937 (3.4 μg/mg mosquito). The mosquitoes were returned to rearing conditions for 24 h after which they were transferred to individual egg laying tubes consisting of a glass tube 21 × 70 mm (Fisher Scientific, Pittsburg, PA) with a piece of coffee filter (Melitta USA, Clearwater, FL) cut to fit the bottom of the tube. The filter was wetted with 150 μl of dH_2_O and the open end of the tube was plugged with a cotton ball. The mosquitoes in their individual egg laying tubes were returned to rearing conditions for an additional 48 h, and the number of eggs laid were counted. Any mosquitoes that died during the 72 h period after blood feeding were excluded from the analysis. Thirty female mosquitoes were used per replicate for each treatment group; each treatment was repeated on four separate broods, giving a total number of individuals studied ranging from 87–113 for each treatment group. For both species, the median number of eggs laid per mosquito was compared using a Kruskal-Wallis ANOVA with a Dunn’s post-hoc analysis (Prism 6, Graphpad Software).

### Honeybee rearing and toxicity experiments

Frames of late-stage honey bee (*A. mellifera*) pupae were taken from four colonies at The Ohio State University Honey Bee Lab in Wooster, OH and maintained in a dark humid incubator at 34 °C (Darwin Chambers Co., St. Louis, MO, model H024) until adult bees emerged. New adults were brushed from frames daily and placed in wooden screen cages (21 × 14 × 12 cm) provisioned with 1:1 (w/w) sucrose in water.

Acute toxicity experiments in adult bees were performed as follows. Twenty-four hours after emergence, adult honey bees in cages were anaesthetized with carbon dioxide, divided into groups of approximately 20 bees and placed in plastic-coated paper cups (177 cm^3^; UNIQ Paper Yogurt Cup, Frozen Dessert Supplies, Gilbert, AZ) covered with cotton cheesecloth. Smaller groups of bees were anaesthetized a second time and dosed on the thoracic notum with 10 μl of VU041 (100 μg/μl) or 10 μl of the vehicle. As a positive control, some bees were treated with 3 μl of bifenthrin (0.1 μg/μl); negative controls consisted of bees treated with 3 μl of solvent. Applications were made using a 20 μl micropipette (Fisherbrand Finnpipette F2, Fisher Scientific, Pittsburgh, PA) with disposable plastic tips. After treatment, bees were returned to the paper cups and provided with sugar syrup in punctured 1.5 ml microcentrifuge tubes. Toxicity to honey bees was recorded at 48 h versus the 24 h used for mosquitoes to ensure VU041 did not cause delayed toxicity due to the larger body size of the bee. Bees showing no movement on inspection at 48 h after treatment were scored as dead. A Fisher’s Exact Test was used to compare the proportion of mortality induced by VU041 and bifenthrin compared to their respective vehicle controls.

## Additional Information

**How to cite this article**: Swale, D. R. *et al.* An insecticide resistance-breaking mosquitocide targeting inward rectifier potassium channels in vectors of Zika virus and malaria. *Sci. Rep.* **6**, 36954; doi: 10.1038/srep36954 (2016).

**Publisher’s note:** Springer Nature remains neutral with regard to jurisdictional claims in published maps and institutional affiliations.

## Supplementary Material

Supplementary Information

## Figures and Tables

**Table 1:** Mean (n = 5) ED_50_ (μg/mg of mosquito) after topical exposure of VU041 with and without synergists, Piperonyl butoxide and S,S,S-tributyl phosphorotrithioate (500 ng/insect) in adult female *An. gambiae*.

Compound(s)	1G3 Strain	1Akron Strain	2Resistance Ratio
VU041	1.8 (0.9–3.1) A	2.8 (2.1–4.1) A	1.5
VU041 + PBO	0.88 (0.3–1.4) B	0.37 (0.2–0.7) B	0.4
VU041 + DEF	1.7 (1.1–2.2) A	N.D.	N.D.
VU730	2.4 (2.1–2.9) A	N.D.	N.D.
VU730 + PBO	0.9 (0.4–1.5) B	N.D.	N.D.
VU937	>10	>10	N.D.
Permethrin*	0.03e-5 (0.02e-5–0.05e-5)	1.0e-3 (0.8e-4–1.3e-3)	33

Mosquitoes were pre-treated with PBO and DEF four hours prior to VU041 treatment. Mosquito strains not labeled by the same letter indicates statistical significance (P < 0.05) of the means as determined by a one-way ANOVA with Tukey’s post-hoc test. N.D. represents no data.

^1^ED_50_, μg/mg (95% confidence limits).

^2^RR: Resistance ratio for G3 = ED_50_ Akron/ED_50_ G3.

^*^Topical ED_50_ values from reference^49^.
