# Supplementary material for: An insecticide resistance-breaking mosquitocide targeting inward rectifier potassium channels in vectors of Zika virus and malaria
Source: Sci Rep. 2016 Nov 16;6:36954. doi: 10.1038/srep36954 (PMC5111108; doi:10.1038/srep36954)
Supplement: Supplementary Information [file srep36954-s1.pdf]

Supplemental Information

An insecticide resistance-breaking mosquitocide targeting inward rectifier potassium channels in vectors of Zika virus and malaria

Authors**:** Daniel R. Swale[1,9], Darren W. Engers[2,4], Sean R. Bollinger[2,4], Aaron Gross[7], Edna Alfaro Inocente[8], Emily Days[5], Fariba Kanga[8], Reed M. Johnson[8], Liu Yang[8], Jeffrey R. Bloomquist[7], Corey R. Hopkins[2,3,4], Peter M. Piermarini[8], Jerod S. Denton[1,2,5,6]

Author Affiliations**:**

Department of Anesthesiology[1], Department of Pharmacology[2], Department of Chemistry[3], Vanderbilt Center for Neuroscience Drug Discovery[4], Vanderbilt Institute of Chemical Biology[5], Institute for Global Health[6], Vanderbilt University Medical Center, Nashville, TN 37232. Department of Entomology and Nematology, University of Florida, Gainesville, FL 32610[7], Department of Entomology, Ohio Agricultural Research and Development Center, The Ohio State University, Wooster, OH 44691[8]. Department of Entomology, Louisiana State University Agricultural Center, Baton Rouge, LA, 70803[9].

Supplemental Methods

Tl^+ flux assay development

Tl^+ flux assays were performed essentially as described previously (1). Briefly, stably transfected T-Rex-HEK-293-*An*Kir1 cells were cultured overnight in 384-well plates in media containing DMEM, 10% dialyzed FBS and 1μg/mL tetracycline to induce channel expression (Fig. S1A). The next day, the cell culture medium was replaced with a dye-loading solution containing assay buffer (Hanks Balanced Salt Solution with 20 mM HEPES, pH 7.3), 0.01% (*w/v*) Pluronic F-127 (Life Technologies, Carlsbad, CA), and 1.2 μM of the thallium-sensitive dye Thallos-AM (TEFlabs, Austin, TX). Following 1 hr incubation at room temperature, the dye-loading solution was washed from the plates and replaced with 20 μL/well of assay buffer. The plates were transferred to a Hamamatsu Functional Drug Screening System 6000 (FDSS6000; Hamamatsu, Tokyo, Japan) where 20 μL/well of test compounds in assay buffer (as prepared below) were added and allowed to incubate with the cells for 20 min. After the incubation period, a baseline recording was collected at 1 Hz for 10 s (excitation 470 ± 20 nm, emission 540 ± 30 nm) followed by a thallium stimulus buffer addition (10 μL/well) and data collection for an additional 4 min. The Tl^+ stimulus buffer contains in (mM) 125 $NaHCO_3$, 1.8 $CaSO_4$, 1 $MgSO_4$, 5 glucose, 12 Tl_2SO_4, 10 HEPES, pH 7.4. For Tl^+ flux assays on Kir2.x, Kir4.1 and Kir6.2/SUR1 expressing cells, the Tl^+ stimulus buffer contained 1.8 mM Tl_2SO_4. To ensure the small-molecule vehicle DMSO had no direct effect on *An*Kir1-dependent Tl+ flux, the assay's tolerance to different doses of DMSO was evaluated (Fig. S1B). The robustness and reproducibility of the assay was determined by comparing Tl^+ flux through tetracycline-induced and tetracycline-uninduced cells (Fig. S1C). The Z' value was calculated as described previously (1), using the following formula:

$$Z' = 1- (3SD_p + 3SD_n)/|mean_p + mean_n|$$

where SD is standard deviation, p and n are control and uninduced flux values respectively.

To compare the effect of DMSO on *An*Kir1-mediated Tl^+ flux, a one-way ANOVA was performed with a Tukey's multiple comparison test. Prism software (GraphPad Software) was used to generate CRC from Tl^+ flux. Half-inhibition concentration (IC_{50}) values were calculated from fits using a four parameter logistic equation.

High-throughput screening

The test compounds were transferred to daughter polypropylene 384-well plates (Greiner Bio-One, Monroe, NC) using an Echo555 liquid handler (Labcyte, Sunnyvale, CA), and then diluted into assay buffer to generate a 2X stock in 0.6% DMSO (0.3% final). For Tl^+ flux assays on Kir6.2/SUR1 expressing cells, test compounds were diluted in assay buffer containing diazoxide (250 μM final) to induce channel activation. Concentration-response curves (CRCs) were generated by screening compounds at 3-fold dilution series (1 nM - 30 μM).

Tl^+ flux data were analyzed as previously described (2, 3) using a combination of Excel (Microsoft Corp, Redmond, WA) with XLfit add-in (IDBS, Guildford, Surrey, UK), OriginPro (OriginLab, Northampton, MA), and GraphPad Prism (GraphPad Software, San Diego, CA, USA) software. Raw data were opened in Excel and each data point in a given trace was divided by the first data point from that trace (static ratio) followed by subtraction of data points from control traces generated in presence of vehicle controls. The slope of the fluorescence increase beginning 5 s after Tl^+ addition and ending 15 s after Tl^+ addition was calculated.

Patch clamp electrophysiology

Patch electrodes were pulled from silanized 1.5 mm outer diameter borosilicate microhematocrit tubes using a P-1000 Flaming/Brown micropipette puller (Sutter Instrument, Novato CA, USA). Electrode resistance ranged from 2-4 MΩ. Whole-cell currents were recorded under voltage-clamp conditions using an Axopatch 200B amplifier (Molecular Devices, Sunnyvale, CA). Electrical connections to the amplifier were made using Ag/AgCl wires and 3 M KCl/agar bridges. Electrophysiological data were collected at 5 kHz and filtered at 1 kHz. Data acquisition and analysis were performed using pClamp 9.2 software (Axon Instruments).

Two-electrode voltage clamp electrophysiology

Defolliculated *Xenopus laevis* oocytes (Ecocyte Bioscience, Asutin, TX) were injected with *Ae*Kir1 or *Ae*Kir2B cRNA (10 ng) and cultured in OR3 media for 3–7 days at 18°C. Electrophysiology experiments were performed exactly as described previously (1, 4). The compositions of the solutions used for electrophysiology experiments are shown in Table S4. When present, VU041 was dissolved in solution *III* to a final concentration of 50 μM (0.05% DMSO). All solutions were delivered by gravity at a flow rate of ~2 ml/min to a RC-3Z oocyte chamber (Warner Instruments, Hamden, CT) using polyethylene tubing, and solution changes were performed with a Rheodyne Teflon 8-way Rotary valve (Model 5012, Rheodyne, Rohnert Park, CA).

In brief, for a given experiment, an oocyte was transferred to the RC-3Z chamber under superfusion (solution *I*). To measure membrane potential (V_m) and whole-cell membrane current (I_m) of the oocyte, it was impaled with two glass microelectrodes backfilled with 3 M KCl (resistances of 0.5–1.5 MΩ). Each microelectrode was bridged to an OC-725 oocyte clamp

(Warner Instruments) and was under the digital control pCLAMP software (Clampex module, version 10, Molecular Devices, Sunnyvale, CA).

The voltage clamp was then turned off during solution changes, and when the oocyte reached a new steady-state V_m (~90 s) the I-V relationship of the oocyte was acquired again. All V_m and I_m values were digitally recorded (Digidata 1440A Data Acquisition System, Molecular Devices) and the resulting I-V plots were generated with the Clampfit module of pCLAMP.

To measure the inhibition of Kir channel activity by VU041, we focused on the maximal inward currents elicited, which occur at the clamp voltage of -140 mV during the voltage-stepping protocol. The background current of an oocyte in solution *II* (low K^+) was subtracted from that in 1) solution *III* (elevated K^+) to calculate the inward current before exposure to VU041 (I_A), and 2) solution *III* with VU041 to calculate the inward current after exposure to a small molecule (I_B). The percent inhibition of I_A by VU041 was calculated by subtracting I_B from I_A and then dividing by I_A.

Chemical synthesis

All NMR spectra were recorded on a 400 MHz FT-NMR DRX-400 FT-NMR spectrometer or 500 MHz Bruker DRX-500 FT-NMR spectrometer. ^{1}H chemical shifts are reported in δ values in ppm downfield with the deuterated solvent as the internal standard. Data are reported as follows: chemical shift, multiplicity (s = singlet, d = doublet, t = triplet, q = quartet, br = broad, m = multiplet), integration, coupling constant (Hz). High resolution mass spectra were recorded on a Waters Q-TOF API-US plus Acquity system with electrospray ionization. Reversed-phase LCMS analysis was performed using an Agilent 1200 system comprising a binary pump with degasser, high-performance autosampler, thermostatted column

compartment, diode-array detector (DAD) and a C18 column. Flow from the column was split to a 6130 SQ mass spectrometer and Polymer Labs ELSD. The MS detector was configured with an electrospray ionization source. Data acquisition was performed with Agilent Chemstation and Analytical Studio Reviewer software. Samples were separated on a ThermoFisher Accucore C18 column (2.6 um, 2.1 x 30 mm) at 1.5 mL/min, with column and solvent temperatures maintained at 45 C. The gradient conditions were 7% to 95% acetonitrile in water (0.1% TFA) over 1.1 minutes. Low-resolution mass spectra were acquired by scanning from 135 to 700 AMU in 0.25 seconds with a step size of 0.1 AMU and peak width of 0.03 minutes. Drying gas flow was 11 liters per minute at a temperature of 350 C and a nebulizer pressure of 40 psi. The capillary needle voltage was 3000 V, and the fragmentor voltage was 100V. Preparative purification was performed on a custom HP1100 purification system (reference 16) with collection triggered by mass detection. Solvents for extraction, washing and chromatography were HPLC grade. All reagents were purchased from Aldrich Chemical Co. and were used without purification.

General procedures for compound synthesis

To a solution of an amine (1 eq.) and pyridine (4 eq.) in DMF (2 mL) was added chloroacetyl chloride (1.3 eq.). After 30 min at rt, the reaction was added to a mixture of EtOAc and water (1:1). The aqueous layer was extracted with EtOAc and the organic extraction was washed with water. The organic extraction was concentrated under reduced pressure to yield the desired α-chloroacetamide.

A solution of the α-chloroacetamide (1 eq.), an amine (1 eq.) and cesium carbonate (1 eq.) in DMF was heated to 150°C for 5 min in a microwave reactor. The solution was filtered (0.45μm) and fractions were separated via reverse-phase HPLC in a gradient of MeCN in water

(0.1%TFA). Fractions were combined and added to water:EtOAc (1:1) and added aq. $NaHCO_3$. The organic layer was collected and solvent was removed on air concentrator. Residue was resuspended in DCM/MeOH and filtered through phase separator into vial yielding the desired final products.

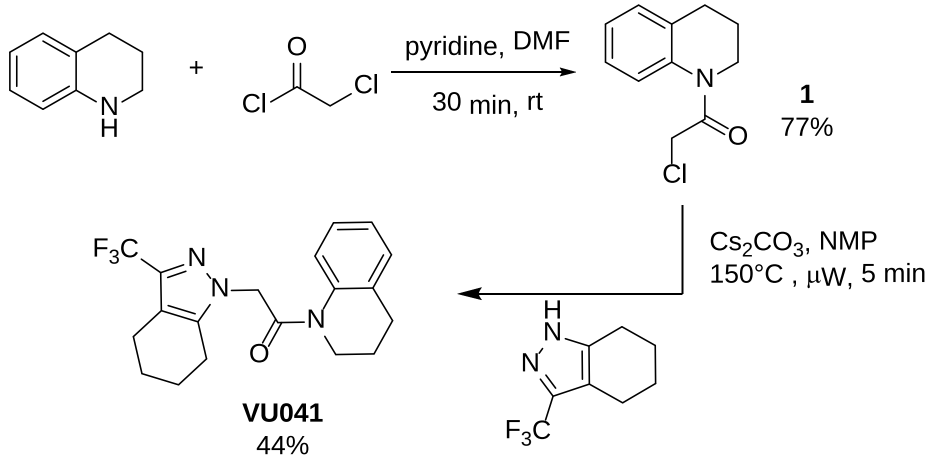

Synthesis of 2-chloro-1-(3,4-dihydro-2H-quinolin-1-yl)ethanone (1)

To a solution of 1,2,3,4-tetrahydroquinoline (0.19 mL, 1.5 mmol) and pyridine (0.50 mL, 6.2 mmol) in DMF (2 mL) was added chloroacetyl chloride (0.16 mL, 2.0 mmol). After 30 min at rt, the reaction was added to a mixture of EtOAc and water (100mL:100mL). The aqueous layer was extracted with EtOAc (100 mL). The organic extraction was washed with water 3X (300 mL). The organic extraction was concentrated under reduced pressure to yield **1** (243 mg, 1.16 mmol, 77% yield). LCMS: 0.90 min, >90% at 215 and 254nm, $[M + H]^+$ = 210.2.

Synthesis of 1-(3,4-dihydroquinolin-1(2H)-yl)-2-(3-(trifluoromethyl)-4,5,6,7-tetrahydro-1H-indazol-1-yl)ethan-1-one (VU041)

A solution of **1** (25 mg, 0.12 mmol), 3-(trifluoromethyl)-4,5,6,7-tetrahydro-1*H*-indazole (22.7 mg, 0.12 mmol) and cesium carbonate (38.8 mg, 0.12 mmol) in DMF (1 mL) was heated to

150°C for 5 min in a microwave reactor. The solution was filtered (0.45μm) and fractions were separated via reverse-phase HPLC in a gradient of MeCN in water (0.1%TFA). Fractions were combined and added to a mixture of water (15 mL) and EtOAc (15 mL). To the mixture was added a saturated solution of $NaHCO_3$ (1 mL). The organic layer was collected and solvent was removed on air concentrator. Residue was resuspended in DCM:MeOH and filtered through phase separator into vial, yielding **VU041** (43.4 mg, 0.0523 mmol, 44% yield). LCMS: R_T = 0.793 min, >95% at 215 and 254nm, $[M + H]^+$ = 364.1.

Lead optimization

Commercial 1,2,3,4-tetrahydroquinoline was treated with chloroacetyl chloride in the presence of pyridine to yield the **1**. Next, the appropriate nitrogen heterocycle was reacted in a microwave reactor with **1** under basic conditions yielding the desired compounds. The SAR is outlined in Tables S1-S2. Our first library was designed to keep the right-hand dihydroquinoline constant and evaluate the left-hand heterocyclic portion (Table S2). If the six-membered ring was aromatized, the compound lost activity against *An*Kir1, **2**. Addition of a carbonyl in the 4-position of the tetrahydroindazole, **3**, also led to an inactive compound. Interestingly, deletion of a nitrogen from **3** brought some activity back into **4** (12.1 μM). One compound (**VU730**, **5**) retained its activity toward *An*Kir1 (IC_{50}=2.4 μM), but lost activity toward Kir2.1 (IC_{50}>30 μM). Expanding the ring system to incorporate a tetrahydroquinoline retained some activity against *An*Kir1 (**6**, 8.0 μM), and deletion of the 6-membered ring of the tetrahydroisoquinoline leaving the unsubstituted pyrazole was unproductive (**7**, inactive). Our next library kept the left-hand trifluoromethyl tetrahydropyrazole (**VU041, 1**) constant while altering the right-hand amide portion of the molecule (Table S3). Moving from the tetrahydroquinoline to the

tetrahydroisoquinoline led to an ~7-fold loss of potency (**8**, 15 μM). Moving to the decahydroquinoline (**9**, 6.7 μM) retained some activity while the regioisomer was inactive (**10**). Moving to the piperidine ring was not productive (**11**); however, adding pendant substitution led to active compounds (phenyl, **12**, 5.6 μM and dimethyl, **13**, 4.5 μM). Addition of oxygen in a benzo[*b*]oxazine structure (**14**, 4.6 μM) retained activity; and like the piperidine scaffold, deletion of the phenyl portion led to an inactive compound (morpholine, **15**, inactive). Addition of a phenyl substitution did not bring back activity, unlike in the piperidine scaffold, **16**. Finally, moving the nitrogen outside of the ring system led to an inactive compound (**17**) and other smaller ring systems were not tolerated, **18**. Compound **18**, VU937 inhibited the *An*Kir1 channel activity by 60-fold less than VU041 with an IC_{50} of 29,670 nM (95% CI: 17,680 – 49,770 nM) (Fig. 1).

Supplemental References

1. Raphemot R, et al. (2014) Discovery and Characterization of a Potent and Selective Inhibitor of *Aedes aegypti* Inward Rectifier Potassium Channels. *PLoS One* 9(11):e110772.
2. Raphemot R, et al. (2011) Discovery, characterization, and structure-activity relationships of an inhibitor of inward rectifier potassium (Kir) channels with preference for Kir2.3, Kir3.x, and Kir7.1. *Front Pharmacol* 2:75.
3. Raphemot R, et al. (2013) Development and validation of fluorescence-based and automated patch clamp-based functional assays for the inward rectifier potassium channel Kir4.1. *Assay and Drug Development Technologies* 11(9-10):532-543.

4. Piermarini PM, et al. (2015) Localization and role of inward rectifier K channels in Malpighian tubules of the yellow fever mosquito *Aedes aegypti*. *Insect Biochem Mol Biol.*

Table S1. Selectivity of VU041 against mosquito and mammalian Kir channels. IC_{50} values were determined using the thallium-flux assay and are expressed as means (*n* = 3). Selectivity ratios (SR) are expressed as: *An*Kir1 IC_{50}/secondary Kir IC_{50}. Mean IC_{50} values were compared by a one-way ANOVA followed by Tukey's posttest using InStat™ (GraphPad Software, San Diego CA, USA). The letters indicate statistical categorization of the means

**Kir Channel**	**IC_{50} (□(r C**	**Selectivity Ratio**
*An*Kir1	2.5 (1.1-3.6) A	-
*Ae*Kir1	1.7 (0.6-2.8) A	0.7
Kir1.1	> 30 B	> 12
Kir2.1	12.7 (10.2-14.3) C	5
Kir4.1	> 30 B	> 12
Kir6.2+SUR1	> 30 B	> 12
Kir7.1	> 30 B	> 12

Table S2. Structure-activity relationship (SAR) on the left-hand portion of VU041.

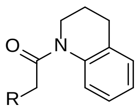

Cmpd	VU#	R	*An*Kir1 IC_{50} (μM)	hKir2.1 IC_{50} (μM)
**1**	VU0048041	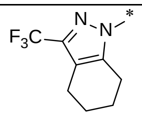	2.3	11.3
**2**	VU0650728	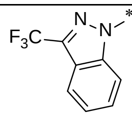	Inactive	N.D.
**3**	VU0650727	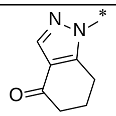	inactive	N.D.
**4**	VU0650729	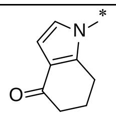	12.1	>30
**5**	VU0650730	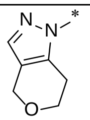	2.4	>30
**6**	VU0657111	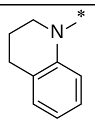	8.0	N.D.
**7**	VU0657112	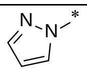	Inactive	N.D.

Table S3. SAR on the right-hand portion of VU041

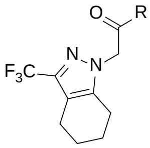

Cmpd	VU#	R	*An*Kir1 IC_{50} (μM)	hKir2.1 IC_{50} (μM)
**1**	VU0048041	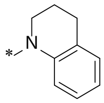	2.3	N.D.
**8**	VU0652933	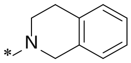	15	N.D.
**9**	VU0652954	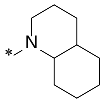	6.7	>30
**10**	VU0652944	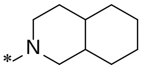	Inactive	N.D.
**11**	VU0652947	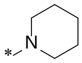	Inactive	N.D.
**12**	VU0652942	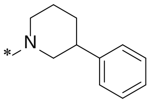	5.6	>30
**13**	VU0652943	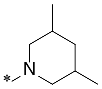	4.5	>30

**14**	VU0652934	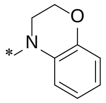	4.6	N.D.
**15**	VU0299695	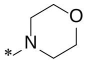	Inactive	N.D.
**16**	VU0652955	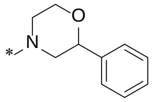	Inactive	N.D.
**17**	VU0652931	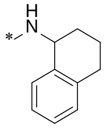	Inactive	N.D.
**18**	VU0652937	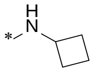	inactive	N.D.

Table S4. Chemical composition of solutions used in *Xenopus* oocyte experiments.

Solution #	I	II	III
NaCl	96	88.5	88.5
NMDG-Cl	0	9.5	0
KCl	2	0.5	10
$MgCl_2$	1.0	1.0	1.0
$CaCl_2$	1.8	1.8	1.8
HEPES	5	5	5

The pH of all solutions was adjusted to 7.5 with NMDG-OH.

The osmolality of each solution was verified to be 190 ± 5 mOsm/kg H_2O by vapor pressure osmometry.

NMDG = N-methyl D-glucamine.

Supplemental Figures

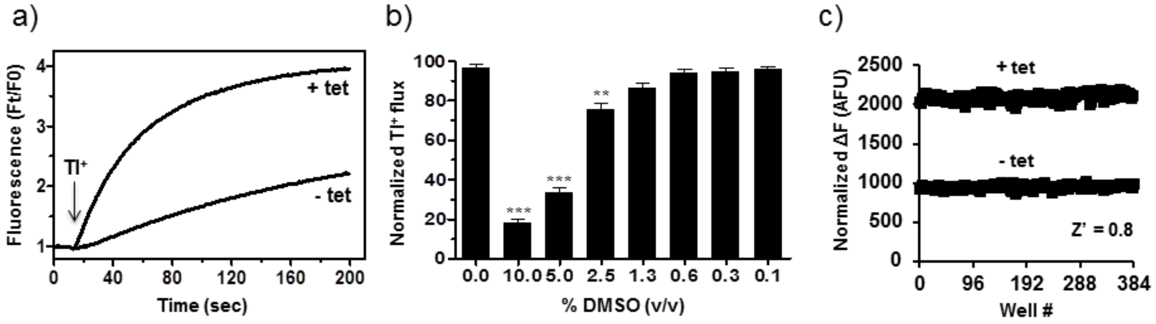

Figure S1. (A) Representative Tl^+-induced changes in Thallos© fluorescence in T-Rex-HEK293-AnKir1 cells cultured overnight with (+Tet) or without (-Tet) tetracycline. The arrow indicates the cell exposure to Tl^+. (B) DMSO concentrations up to 1.3% v/v DMSO have no effect on Tl^+ flux through *An*Kir1. Data are means (n = 3) with error bars representing SEM. One-way ANOVA P: 0.0001, and asterisks (**, ***) indicate P<0.01 or P<0.001 respectively, when compared to 0% DMSO (Tukey's posttest). (C) Representative checkerboard analysis using uninduced cells or induced cells. The mean peak fluorescence amplitude of each sample population is indicated with a solid line and alternating samples for induced and uninduced are graphed as individual points. The mean SD Z' calculated over 6 plates on 3 separate days was 0.8.

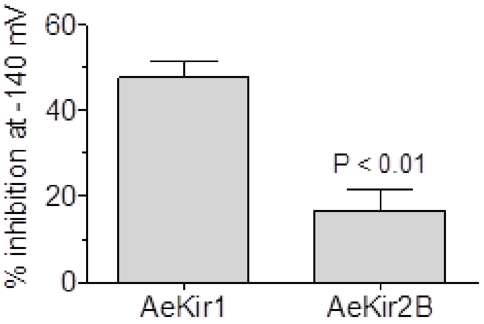

Figure S2. Percent inhibition of inward K^+ currents by VU041 in *Xenopus* oocytes when membrane potential was clamped at -140 mV. Oocytes were heterologously expressing *Ae*Kir1 (n = 4) or *Ae*Kir2B (n = 3). P-value indicates statistical significance as determined by an unpaired t-test (Prism 6, Graphpad Software).

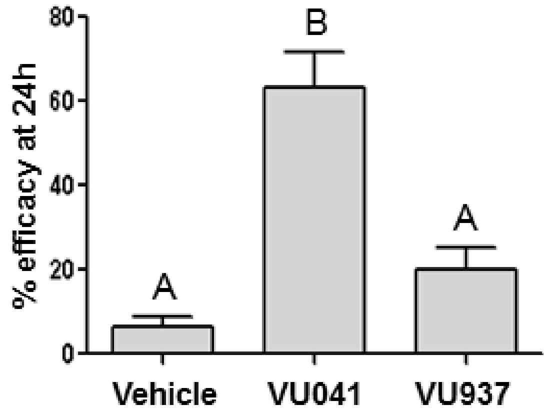

Figure S3. Toxicity of VU041 and VU937 (3.24 µg/mg mosquito for each compound) in adult female *Ae. aegypti* (LVP) compared to vehicle controls 24 h after topical exposure. n = 6 trials of 10 mosquitoes for each treatment. Upper-case letters indicate statistical categorization of the means as determined by a one-way ANOVA with a Newman-Keuls posttest ($P < 0.05$) (Prism 6).
